# Relationship between epicardial adipose tissue, coronary artery disease and adiponectin in a Mexican population

**DOI:** 10.1186/1476-7120-12-35

**Published:** 2014-09-08

**Authors:** Teresa G Yañez-Rivera, Manuel A Baños-Gonzalez, Jorge L Ble-Castillo, Manuel E Torres-Hernandez, Jorge E Torres-Lopez, Gabriela Borrayo-Sanchez

**Affiliations:** 1 Departamento de Cardiología, Hospital General de Zona 46, Instituto Mexicano del Seguro Social (IMSS), Villahermosa, Tabasco, Mexico; 2 Departamento de Cardiología, Hospital Regional de Alta Especialidad “Dr. Juan Graham Casasús”, Villahermosa, Tabasco, Mexico; 3 Centro de Investigación y Posgrado, DACS, Universidad Juárez Autónoma de Tabasco (UJAT), Villahermosa, Tabasco, Mexico; 4 Coordinación de Investigación, Centro Médico Nacional Siglo XXI, IMSS, Mexico City, México

**Keywords:** Epicardial adipose tissue, Coronary artery disease, Echocardiography, Adiponectin

## Abstract

**Background:**

The amount of epicardial adipose tissue (EAT) around the heart has been identified as an independent predictor of coronary artery disease (CAD), potentially through local release of inflammatory cytokines. Ethnic differences have been observed, but no studies have investigated this relationship in the Mexican population. The objective of the present study was to evaluate whether a relationship exist between EAT thickness assessed via echocardiography with CAD and adiponectin levels in a Mexican population.

**Methods:**

We studied 153 consecutive patients who underwent coronary angiography and transthoracic echocardiography (TTE). EAT thickness on the free wall of the right ventricle was measured at the end of systole from parasternal long and short axis views of three consecutive cardiac cycles. Coronary angiograms were analyzed for the presence, extent and severity of CAD. Serum adiponectin, lipids, glucose, C-reactive protein and fibrinogen were determined.

**Results:**

EAT thickness was greater in patients with CAD than in those without CAD from both parasternal long (5.39 ± 1.75 mm vs 4.00 ± 1.67 mm p < 0.0001) and short-axis views (5.23 ± 1.67 vs 4.12 ± 1.77, p = 0.001). EAT thickness measured from parasternal long and short-axis showed a statistically significant positive correlation with age (r = 0.354, p < 0.001; r = 0.286, p < 0.001 respectively), and waist circumference (r = 0.189, p = 0.019; r = 0.217, p = 0.007 respectively). A significant negative correlation between EAT thickness from the parasternal long axis with cholesterol-HDL was observed (r = -0.163, p = 0.045). No significant correlation was found between epicardial fat thickness and serum adiponectin or with the severity of CAD.

**Conclusions:**

EAT thickness was greater in patients with CAD. However, no correlation was observed with the severity of the disease or with serum adiponectin levels. EAT thickness measured by echocardiography might provide additional information for risk assessment and prediction of CAD.

## Background

Obesity is the most prevalent risk factor for coronary artery disease [1]. Regional body adipose tissue distribution, rather than total body adiposity, is a known marker for cardiovascular risk [2]. A variety of pro and anti-inflammatory mediators are expressed and secreted from adipose tissue and have been demonstrated to participate in the various stages of atherogenesis [3]. Adiponectin is exclusively identified in mature adipocytes and decreased plasma levels are associated with atherosclerosis [4]; adiponectin levels are inversely correlated with the amount of visceral adipose tissue and may serve as a predictive factor for cardiovascular risk [5,6]. Epicardial adipose tissue (EAT) locally expresses adiponectin protein, with significantly lower levels in patients with coronary artery disease (CAD) [7-9].

EAT is considered as visceral fat deposited around the heart, particularly around epicardial coronary vessels. Because of its proximity to the myocardium and absence of fascia, epicardial fat may directly affect the coronary arteries and myocardium through paracrine actions of locally secreted adipocytokines and other bioactive molecules, contributing to the development of CAD [7,10,11].

Recently, a meta-analysis demonstrated that EAT thickness is an effective marker for predicting CAD [12]. However, several studies have shown that race can affect the distribution of intra-abdominal adipose tissues [13], and also participate in the observed differences in EAT thickness [14,15]. Mexico exhibits the highest obesity prevalence in the world, even so, to our knowledge there are no published studies on EAT thickness related to CAD and adiponectin. Thus, the present study was designed to assess whether an association exists between epicardial adipose tissue measured by transthoracic echocardiography with CAD and serum adiponectin in a Mexican population.

## Methods

### Patients

From February 2011 to June 2013, we conducted a cross-sectional study on all patients admitted for elective coronary angiography for suspected coronary artery disease.

For all subjects, clinical data and anthropometric measures were recorded. Individuals were considered as type 2 diabetic if they had been previously diagnosed, were receiving hypoglycemic treatment and/or insulin, or had a fasting glucose level of >126 mg/dl on two or more occasions. Hypertension was defined as systolic blood pressure ≥140 mmHg, diastolic blood pressure >90 mmHg or if previously diagnosed or receiving a previously established antihypertensive therapy. Hyperlipidemia was defined as a total cholesterol (TC) ≥200 mg/dl, Low-density lipoprotein cholesterol (LDL-cholesterol) greater than ≥160 mg/dl, triglycerides (TG) ≥150 mg/dl and/or a previous diagnosis or treatment. Decreased levels of high-density lipoprotein cholesterol (HDL-C) were defined as <40 mg/dl. Patients were considered smokers if they had smoked five or more cigarettes a day and had suspended this habit for longer than one year. Body mass index (BMI) was calculated by a standard formula [weight (kg)/height (m)^2^]. Central obesity was considered if the waist circumference was ≥90 cm in men and ≥80 cm in women [16]. Patients with more than three of five criteria based on the International Diabetes Federation (IDF) were considered to have metabolic syndrome (MS). Patients were excluded if they had any of the following: chronic renal and/or hepatic disease, history of prior revascularization, patients with pericardial effusion and whose transthoracic echocardiographic imaging was inadequate for the measurement of epicardial fat thickness. Mexican Social Security Institute (IMSS) Research Committee approved the study protocol, and all patients gave written informed consent.

### Laboratory measurements

Blood samples were obtained after a fast of at least 8 h. Serum and plasma were immediately distributed in aliquots and frozen at -70°C for less than six months. The samples were analyzed in blocks to reduce inter-assay variability. Glucose concentrations, plasma lipid profile and high sensitivity C-reactive protein (hsCRP) were measured using the Architect Clinical Chemistry Autoanalyzer System from Abbott Clinical Chemistry (Chicago, IL, USA). The assay reliability of these parameters was < 5% coefficient of variation. Fibrinogen determination was performed using ACL Elite Pro equipment with the Clauss method. Plasma concentrations of adiponectin were determined using a commercially available immunoassay kit SPI-bio Bertin Pharma (Montigny le Bretonneux, France) and the values were expressed in μg/ml.

### Evaluation of epicardial adipose tissue

Two-dimensional transthoracic echocardiography (Philips iE33, with 3.5 MHz transducer) in the left lateral decubitus position was performed. Measurements were performed by a cardiologist who was unaware of angiographic data. Epicardial adipose tissue was identified as echo-free space in the pericardial layers on the two-dimensional echocardiography and its thickness was measured perpendicularly on the free wall of the right ventricle at the end of systole, repeated over three cardiac cycles. Two standard echocardiographic windows, parasternal long-axis and short-axis, were used. A pre-specified anatomical reference for each echocardiographic window was considered as a standard measurement point in order to be consistent with other studies. For the parasternal long axis view, the anatomical reference was the aortic annulus. Measurements were performed at the point on the right ventricular free wall in the midline with the ultrasound beam, perpendicular to the aortic annulus. For the parasternal short axis view, epicardial adipose tissue thickness was measured on the right ventricular free wall, 2 cm from the ventricular septum. We calculated an average value of three cardiac cycles for each echocardiographic window for statistical analysis. Echocardiographic measurement of epicardial thickness was defined as an epicardial thickness >1 mm in at least one acoustic window. The reliability of transthoracic echocardiography for measuring EAT thickness, has been confirmed by its good correlation with magnetic resonance imaging [17].

### Coronary artery occlusion assessment

Selective coronary angiography was performed using femoral access in all patients. Patients were classified as CAD+, defined as >50% stenosis in at least one major coronary artery, or CAD - with no angiographic evidence of significant coronary artery occlusion. Severity of coronary atherosclerosis was classified as 1-, 2-, or 3-vessel disease according to the number of stenotic major coronary arteries (left anterior descending artery, circumflex artery and right coronary artery).

### Statistical analysis

We used descriptive statistics with mean ± SD or median with interquartile range values in accordance with their distribution. Student’s *t*-test or Mann–Whitney tests were performed to compare the differences between continuous variables according to their distribution. Significant differences between categorical variables were evaluated using a Chi-square test. The Pearson or Spearman correlation tests were used to evaluate an association of EAT and adiponectin with other quantitative variables. Multiple logistic regression analysis was used to estimate the association between variables and the presence or absence of CAD. In order to determine the predictive value of EAT thickness and adiponectin levels multiple logistic regression analysis was applied to these variables as well as the other factors including conventional clinical variables. In this model, those variables that showed p values < 0.05 in the univariate analysis were included. Odds ratios and 95% confidence intervals (CIs) for CAD + vs CAD- were calculated. A value of p < 0.05 was considered to be statistically significant. Analyses were performed using SPSS, version 15 (Chicago, IL, USA).

To assess the reproducibility of echocardiographic measurements of epicardial adipose tissue, 24 patients were randomly selected for off-line analysis by two observers who were unaware of clinical and angiographic data and inter-observer correlation coefficients were calculated. In the same group of patients, echocardiographic measurements were repeated two days later to calculate intra-observer correlation coefficients.

## Results

### Clinical characteristics

Clinical and laboratory data of 153 patients with or without CAD are summarized in Table 1. Our patient cohort consisted of 119 subjects with CAD (77.7%) and 34 subjects without CAD (22.2%). Male gender was more predominant in the CAD + group (p < 0.001). Compared to those without significant stenosis, patients with CAD had higher waist-hip ratio (0.98 ± 0.06 vs 0.917 ± 0.08, p < 0.001), showed a higher C-reactive protein (3.01 ± 5.65 vs.1.08 ± 2.08 mg/dl, p = 0.002) and a lower HDL cholesterol level (42.35 ± 11.35 vs. 35.81 ± 8.94 mg/dl, p = 0.003). The ejection fraction measured by echocardiography was similar between the CAD- and CAD + groups.

**Table 1:** Clinical and laboratory characteristics in patients with and without coronary artery disease

	**CAD- (n = 34)**	**CAD + (n = 119)**	**p value**
Age (years)	59.18 ± 12.25	61.76 ± 10.05	0.210
Male gender, n (%)	14 (41.1)	98 (82.3)	<0.001
BMI (kg/m^2^)	30.74 ± 5.90	28.92 ± 3.87	0.198
Central obesity n (%)	33 (97.0%)	118 (99.1%)	0.791
WC (cm)	100.97 ± 11.81	103.47 ± 8.81	0.256
Waist-hip ratio	0.92 ± 0.08	0.98 ± 0.06	<0.001
Smoking, n (%)	6 (17.6)	64 (53.7)	<0.001
Diabetes mellitus, n (%)	13 (38.2)	65 (54.6)	0.093
Hypertension, n (%)	26 (76.5)	85 (71.4)	0.564
Hyperlipidemia, n (%)	24 (70.5)	85 (71.4)	0.924
Metabolic syndrome n (%)	29 (85.2)	111 (93.2)	0.141
FPG (mg/dl)	104.0 (95.00-119.25)	119.5 (97.00-152.25)	0.076
Total cholesterol (mg/dl)	175.97 ± 41	164.94 ± 48.55	0.231
LDL-C (mg/dl)	103.85 ± 33.58	91.63 ± 37.33	0.088
HDL-C (mg/dl)	42.35 ± 11.35	35.81 ± 8.94	0.003
TG (mg/dl)	141 (106.5-203.5)	151 (105.0-245.0)	0.353
hsCRP (mg/dl)	1.08 ± 2.08	3.01 ± 5.65	0.002
Adiponectin (μg/ml)	11.66 ± 6.77	8.88 ± 4.64	0.039
Fibrinogen (mg/dl)	547.0 (469.0-660.0)	591.5 (480.5-744.0)	0.090

Data are expressed as number and % of patients, mean ± SD or median (25^th^, 75^th^ percentiles). CAD – Coronary artery disease; BMI – Body mass index; WC – Waist circumference; FPG – fasting plasma glucose; LDL-C – low-density lipoprotein cholesterol; HDL-C – high-density lipoprotein cholesterol; TG – triglycerides; hsCRP – high sensitivity C-reactive protein. Central obesity was defined as increased waist circumference.

### EAT thickness and coronary artery disease

Inter-observer and intra-observer correlation coefficients of EAT thickness measurements were 0.96 and 0.94 respectively, indicating excellent reproducibility of echocardiographic measurements. Mean values of epicardial adipose tissue thickness from the parasternal long and short axes views in all patients were 5.08 ± 1.82 and 4.99 ± 1.75 mm, respectively. Forty-eight (31.4%) patients had single vessel, 36 (23.5%) two vessels and 35 (22.9%) had three vessels with >50% of stenosis. Table 2 shows the echocardiographic data. EAT was thicker in patients with CAD than in those without CAD in both the parasternal long axis (5.39 ± 1.75 vs 4.00 ± 1.67 mm, p < 0.0001) and short axis (5.23 ± 1.67 vs 4.12 ± 1.77 mm, p = 0.001) views.

**Table 2:** Echocardiographic findings in patients with and without coronary artery disease

	**CAD- (n = 34)**	**CAD + (n = 119)**	**p value**
EAT PLX (mm)	4.00 ± 1.67	5.39 ± 1.75	<0.0001
EAT PSX (mm)	4.12 ± 1.77	5.23 ± 1.67	0.001

EAT – epicardial adipose tissue; PLX – parasternal long-axis view, PSX – parasternal short-axis view.

### Association of epicardial adipose tissue with various clinical, biochemical and angiographic variables

Table 3 shows the correlations of EAT with clinical, biochemical and angiographic variables. EAT thickness measured from the long and short axes showed a significant positive correlation with age (r = 0.354, p < 0.001 and r = 0.286, p < 0.001 respectively); with waist circumference (r = 0.214, p = 0.008 and r = 0.234, p = 0.004); with waist-hip ratio (r = 0.189, p = 0.019 and r = 0.217, p = 0.007); with waist-height ratio (r = 0.243, p = 0.002 and r = 0.267, p = 0.001). EAT thickness was inversely correlated with the level of high-density lipoprotein cholesterol (r = -0.163, p = 0.045). Eighty-five percent of subjects in the CAD- group and 92% in the CAD + group had MS (Table 1). No significant differences were observed in EAT thickness between patients with MS and without MS in the parasternal long axis (5.05 ± 1.83 vs 5.38 ± 1.74 mm, p = 0.539) and parasternal short axes (4.99 ± 1.75 vs 4.97 ± 1.82 mm, p = 0.970), respectively. There was no significant correlation between echocardiographic epicardial adipose tissue thicknesses and the number of stenotic vessels.

**Table 3:** Univariate correlations between cardiac adipose tissue thickness and clinical variables

	**EAT PLX**	**p value**	**EAT PSX**	**p value**
	***r***		***r***	
Age	0.354	<0.001	0.286	<0.001
Height	-0.004	0.958	0.000	0.997
Weight	-0.023	0.780	-0.004	0.963
Body surface area	-0.009	0.911	-0.003	0.972
BMI	0.049	0.552	0.070	0.389
Waist	0.214	0.008	0.234	0.004
Hip	0.097	0.231	0.107	0.187
WHR	0.189	0.019	0.217	0.007
WHeR	0.243	0.002	0.267	0.001
Total cholesterol	-0.008	0.919	-0.057	0.481
HDL-C	-0.163	0.045	-0.139	0.087
LDL-C	-0.032	0.637	0.096	0.245
Glucose	0.118	0.148	0.060	0.462
hsCRP	0.125	0.124	0.110	0.176
Fibrinogen	0.132	0.107	0.137	0.094
Adiponectin	-0.092	0.257	-0.073	0.368
Number of stenotic vessels	0.004	0.963	0.028	0.763

### Relationship between adiponectin, CAD and epicardial adipose tissue

Serum adiponectin levels were significantly lower in patients with CAD (11.66 ± 6.77 vs 8.88 ± 4.64 μg/ml, p = 0.039). However, no significant correlations were found between EAT thickness obtained from the long- or short-axes and serum adiponectin (Table 3). Moreover, adiponectin levels in this sample were not a predictor of CAD (Table 4).

**Table 4:** Multiple logistic regression analysis for the prediction of significant coronary artery disease

	**OR (95% CI)**	**p value**
**Male**	14.182 (3.877-51.876)	<0.0001
**WHR**	0.952 (0.921-0.985)	0.004
**Smoking**	3.385 (1.064-10.767)	0.039
**HDL-C**	0.992 (0.943-1.044)	0.761
**hsCRP**	1.165 (0.979-1.385)	0.085
**Adiponectin**	0.977 (0.898-1.063)	0.583
**EAT thickness**	1.903 (1.366-2.653)	<0.0001

WHR – waist-hip ratio; HDL-C – high-density lipoprotein cholesterol; hsCRP – high sensitivity C-reactive protein; EAT – epicardial adipose tissue.

### Epicardial adipose tissue as a predictor of coronary artery disease

Table 4 shows the results of the multivariate logistic regression analysis. Those variables that showed significant differences in the univariate analysis were selected for the multivariate analysis for the prediction of CAD. EAT thickness was found to be an independent predictor of obstructive CAD in addition to the well-known CAD risk factors such as male gender, C-reactive protein and low HDL cholesterol.

## Discussion

This study indicates that in a Mexican population, EAT thickness is associated with CAD. Multiple logistic analyses of several CAD risk factors suggested that EAT is an independent factor associated with CAD. This finding is consistent with others reports in different populations. Ahn et al. [18], reported this relationship in a subset of Korean patients. A recent meta-analysis showed that EAT may be an effective marker for the prediction of coronary heart disease [12]. In our study, mean EAT thickness in patients with coronary heart disease was 5.2 mm, similar to values reported by Mustelier et al. [19], in another Hispanic population. This value is lower than others found in Japanese and USA populations [20,21], but higher than in the Korean population [18].

EAT thickness may be different according to ethnic group. Several studies have shown that ethnicity affects the distribution of visceral adipose tissue. Willens et al. [14], showed that non-Hispanic white males have larger epicardial fat thickness than African-American men, independently of age, weight, and BMI. These differences might be attributed to inherited and nongenetic factors. However, the identities of genes that underlie population variation in adipose tissue development in humans are poorly understood.

The prevalence of obesity has increased worldwide, including in Mexico. Data from the national health and nutrition survey conducted in 2012 showed that obesity affects 7 in 10 Mexican adults [22]. This condition is a prominent risk factor for the development of chronic diseases, such as CAD. Obesity has a strong genetic contribution, in addition to already known environmental and nutritional factors. Our results showed that EAT thickness was significantly correlated with abdominal circumference, but not with BMI. Previous authors have shown similar findings [21]. A partial explanation is that BMI may not predict total adiposity. Although it is known that there are differences in the fat distribution between males and females, in this study a gender analysis was not possible because there were a limited number of women included in the CAD group.

In the present study, no relationship was observed between EAT thickness and the extent of CAD. This result agrees with previous findings from Mustelier et al. [19], but is in contrast with others [18,23]. The lack of association in this study could be partially attributed to the fact that EAT measurements are not performed at a specific anatomical site where the coronary stenosis is located. Moreover, a larger sample size could be needed in order to find this relationship. In this line, the extent of CAD could be related with other risk factors or with the existence of other metabolic disturbances.

Mechanisms that promote the development of CAD through epicardial fat are not completely clear. The role of epicardial fat as a metabolically active organ with paracrine or endocrine functions is the most accepted. Previous studies have shown that adipose tissue, particularly visceral fat, express numerous genes related to adipokine production, which have important roles in the development of atherosclerosis in obese patients [7,24]. Epicardial fat located near to the coronary tree could facilitate the paracrine effects of epicardial adipokines, as part of the pathogenesis of CAD [11,25].

Few studies have evaluated the relationship between EAT thickness and serum adiponectin levels. Yun et al. [26], found a significant inverse relationship between EAT thickness and plasma adiponectin levels in patients with angina. Our data show that patients with CAD have significantly lower adiponectin levels compared to non-CAD subjects, but a significant correlation between EAT thickness and adiponectin was not observed. Furthermore, multivariate analyses did not show adiponectin as an independent variable for CAD risk.

While some studies have related epicardial fat with the presence of inflammatory markers, a direct relationship between these and EAT thickness or CAD has not been demonstrated. Iacobellis et al. [12], reported that protein expression of adiponectin from EAT in patients with extensive CAD was significantly lower than non-CAD subjects; however, if the local adiponectin is produced in EAT rather than systemic influences on coronary heart disease is still debatable [27,28]. Further studies are needed in order to clarify the relation of adiponectin with coronary atherosclerosis.

Imaging studies demonstrated good accuracy for the quantification of adipose tissue in various body compartments, with a good correlation [15]. Transthoracic echocardiography provides a reliable measurement of epicardial adipose tissue thickness. This method has been validated with magnetic resonance imaging [17], which is currently the gold standard method and a widely accepted reference standard to measure visceral adiposity.

Echocardiography as a non-invasive method that is low in cost and widely available could be routinely performed in high-risk patients for the evaluation of epicardial adipose tissue and predicting CAD.

To the best of our knowledge, this is the first study to evaluate an association between EAT thickness, CAD and adiponectin in a Mexican population. Future studies are required to determine reference ranges and cut-off points for EAT thickness measured by echocardiography in Mexican population.

### Study limitations

The present study has several limitations. 1. This cross-sectional study cannot determine causal relationships between studied variables 2. The sample size is modest for association studies. In the studied population, most patients who underwent coronary catheterization have a high risk for CAD, but only a few patients had normal coronary arteries, so we had an unbalanced sample. However, coronary angiography is an invasive study with risks of complications, only indicated in patients with suspected ischemic heart disease and it would not have been ethical to perform it in healthy patients.

## Conclusions

In this study, we observed an association of epicardial fat thickness measured by echocardiography with atherosclerotic CAD, but not with the degree of severity of the disease, or with serum levels of adiponectin. Echocardiography may provide additional information for predicting the risk of CAD in Mexican population.

## Competing interests

The authors declare that they have no competing interests.

## Authors’ contributions

TGYR carried out the ultrasound examinations and was involved in study design and drafting. MABG helped to perform the statistical analysis and draft the manuscript. JLBC was involved in study design and drafted the manuscript. METH carried out the angiography readings and was involved in drafting. JETL participated in the design and helped to draft the manuscript. GBS were involved in statistical analysis and drafting. All authors contributed equally. All authors read and approved the final manuscript.
